# The Role of Ethanol Ablation of the Vein of Marshall in the Era of Pulsed Field Ablation

**DOI:** 10.3390/jcm15051848

**Published:** 2026-02-28

**Authors:** Muazzum M. Shah, Aman Chugh

**Affiliations:** Section of Cardiac Electrophysiology, Division of Cardiology, University of Michigan, Ann Arbor, MI 48109, USA

**Keywords:** atrial fibrillation (AF), vein of Marshall (VOM), ligament of Marshall (LOM), ethanol instillation in the vein of Marshall (EIVOM), mitral isthmus (MI), pulsed-field ablation (PFA)

## Abstract

Atrial fibrillation (AF) is the most common sustained arrhythmia worldwide and is associated with significant morbidity, mortality, and health care expenditure. Catheter ablation is the most effective treatment strategy to decrease AF burden, a measure strongly correlated with significant clinical outcomes. For the last two decades, pulmonary vein isolation (PVI) has remained the cornerstone of the ablation procedure, with single procedure success rates as high as 90% in patients with paroxysmal AF. These favorable outcomes have not translated to patients with persistent AF, who harbor more atrial remodeling and who may require ablation beyond PVI. While most PVI-adjunctive ablation strategies have not survived the rigor of randomized control trials, in 2020, VENUS showed that adjunctive ethanol instillation into the vein of Marshall (EIVOM) improved clinical outcomes in patients with persistent AF. Since VENUS, three other randomized trials have consistently shown a benefit of adjunctive EIVOM, though utilization of this technique remains low due to a multitude of factors. In parallel to the mounting evidence supporting EIVOM, pulsed-field ablation (PFA) has revolutionized the landscape and has called into question the ongoing role of EIVOM. This review examines the electrophysiologic significance of the VOM and summarizes the clinical evidence supporting adjunctive EIVOM in the era of PFA.

## 1. Introduction

Atrial fibrillation (AF) is the most common sustained cardiac arrhythmia and is associated with significant morbidity, mortality, and health care expenditure [1,2,3]. Catheter ablation is the most effective treatment strategy to decrease AF burden, a marker strongly correlated with important clinical outcomes [4,5]. For the last two decades, pulmonary vein isolation (PVI) has remained the cornerstone of the ablation procedure, with success rates reported as high as 90% in patients with paroxysmal AF [6]. However, these favorable results have not translated to patients with persistent AF, who harbor more atrial remodeling and may require extra PV ablation [7]. These suboptimal results have propelled intense investigation of multiple PVI-adjunctive ablation targets in patients with persistent AF, with mixed results [8,9,10].

While most PVI-adjunctive ablation strategies have not been reproducibly successful, in 2020, Valderrábano et al. reported results of the Vein of Marshall Ethanol for Unablated Persistent AF (VENUS) trial [11]. This multicenter randomized control trial demonstrated that instillation of alcohol into the vein of Marshall (VOM)—an embryological remnant of the left superior vena cava that harbors neural elements and myocardial epicardial connections and colocalizes with the lateral mitral isthmus (MI)—led to improvement in outcomes in patients with persistent AF. Since then, three other randomized trials and a meta-analysis of these trials have confirmed these positive findings, though at a cost of increased procedural time, fluoroscopy use, and overall complications [12,13,14,15].

Coincident with the mounting evidence supporting ethanol infusion into the vein of Marshall (EIVOM), the advent of pulsed field ablation (PFA) has disrupted the field of AF ablation. Rather than using thermal injury to cause cell death, PFA uses ultra-short duration high-voltage electrical pulses to cause irreversible electroporation in cell membranes. Studies have shown this modality to be non-inferior to thermal ablation with incremental advantages over thermal ablation in procedural efficiency and safety [16,17,18,19]. This evolving landscape raises the question of the ongoing role of EIVOM in the era of PFA. In this review, we examine the anatomy and electrophysiologic significance of the VOM, summarize the clinical evidence supporting adjunctive EIVOM in patients with AF, and consider the mechanistic and therapeutic limitations of current-generation PFA systems that may warrant ongoing use of EIVOM.

## 2. Anatomic and Electrophysiologic Significance of the Ligament of Marshall

### 2.1. Anatomy

Previous reviews have comprehensively summarized the anatomic and electrophysiologic relevance of the ligament of Marshall (LOM) [20,21]. First described by surgeon and anatomist John Marhsall in 1850, the ligament of Marshall (LOM) is a neurovascular embryological remnant of the left anterior cardinal vein (the precursor of the left superior vena cava) that is made up of the left atrial (LA) oblique vein (the VOM), myocardial fibers insulated in fibrofatty tissue (the Marshall bundle [MB]), and autonomic nerves [22,23]. The LOM lies on the epicardial surface of the LA, in the recess between the left atrial appendage (LAA) and the left pulmonary veins (PVs), along the course of the left lateral ridge. A component of the LOM, the VOM drains the posterior and posterolateral wall of the LA and courses with the ligament until inserting into the coronary sinus (CS) just proximal to the valve of Vieussens. Notably, the VOM colocalizes with the lateral MI between the left PVs and the mitral annulus.

### 2.2. Electrophysiological Significance of the Ligament of Marshall

Anatomic studies of adult human hearts have demonstrated muscular connections between the LOM and CS and posterior LA proximally as well as the left PVs distally [24,25,26,27,28]. In a study of 72 patients undergoing AF ablation and in whom MB potentials were recorded (either via direct cannulation of the VOM or via epicardial recording via a subxiphoid approach), Han et al. demonstrated that most patients had multiple connections between the MB and the LA [29]. The MB can thus be conceptualized as an electroanatomic conduit between these structures and has been implicated in the pathogenesis and maintenance of AF and atrial tachycardias (ATs)—both as a source of focal ectopic beats and by participation in micro/macro-reentrant ATs including perimitral AT, a common form of post-ablation AT [20,30,31,32,33].

In addition to providing epicardial connections to the LA and CS, immunohistochemical staining has shown that the LOM is also richly innervated by both sympathetic and parasympathetic nerve fibers [24,25,34]. Furthermore, animal and clinical studies have shown that high-frequency stimulation of the LOM can induce AF and that chemical ablation of the VOM blunts vagally induced atrial ERP shortening, explaining its antifibrillatory effect. These findings highlight the potential neuromodulatory role of the LOM/VOM in initiating and perpetuating atrial arrhythmias [27,35,36,37].

Finally, as mentioned, the VOM courses epicardially over the lateral MI between the left PVs and CS. This localization affords the opportunity to target the epicardial component of MI-dependent atrial flutter, the most common macro-reentrant arrhythmia following ablation of persistent AF. Linear ablation of the lateral MI has been previously shown to improve outcomes in patients with AF [38,39,40,41]. Knecht et al. previously demonstrated that MI ablation, as part of a step-wise ablation strategy, contributed to acute termination of AF as well as improved arrhythmia-free survival long term, particularly when complete linear block was achieved [42]. However, rates of durable bidirectional block across the MI with conventional ablation are low due to a multitude of factors including tissue thickness, epicardial connections, coronary blood flow, and adipose tissue deposition. Importantly, incomplete block has also been associated with pro-arrhythmia [43,44,45,46]. In addition to reducing the rate of perimitral ATs, the basis for the salutary effect of MI block probably has to do with rapid electrical activity at the MI. In many patients with persistent AF, the most rapid left atrial electrical activity is found at the lateral LA, specifically the LA appendage (LAA) [47]. The rate of activation at the lateral MI often approximates that of the LAA.

## 3. Clinical Evidence Supporting Ethanol Infusion in the Vein of Marshall

Given its specialized electrophysiological properties, evidence for its neuromodulatory role in atrial arrhythmogenesis, and its strategic location, it is no surprise that the VOM has emerged as a therapeutic target for AF/AT. In 2009, Valderrábano et al. reported a technique of retrograde balloon cannulation and ethanol infusion of the VOM in dogs which resulted in localized ablation of the lateral MI and caused regional denervation [48]. Subsequent human studies have demonstrated that EIVOM creates a localized lesion facilitating mitral block as well as left PV isolation, abolishes AF triggers, results in neuromodulation of atrial tissue, and have established the safety profile and feasibility of such an approach [20,27,37,49,50].

This set the stage for the Vein of Marshall Ethanol in Untreated Persistent AF (VENUS) trial, published in 2020 [11]. This multicenter randomized clinical trial of 343 patients evaluated whether conventional radiofrequency (RF) ablation with adjunctive EIVOM improved outcomes in patients undergoing de novo ablation of persistent AF compared to conventional RF ablation alone. The primary outcome was freedom from AF or AT lasting longer than 30 s after a 3-month blanking period without use of an anti-arrhythmic drug judged by monitoring at 6 and 12 months. All patients underwent PVI, and >95% of patients underwent additional ablation beyond PVI in both groups. In the intention-to-treat analysis, 49.2% of the patients in the intervention group remained free of AF/AT compared with 38% in the control group (between-group difference 11.2%; *p* = 0.04). Importantly, a secondary analysis of VENUS showed that the greatest effect of EIVOM was seen in patients in whom MI block was demonstrated (primary outcome achieved in 54.3% of the intervention group vs 37.0% of the control group; between-group difference 17.3%; *p* = 0.01) [44]. In contradistinction, there was no difference in the primary outcome in patients who did not achieve MI block (34.0% in the intervention group vs 37.0% in control group; between-group difference −3.0%; *p* = 0.58), suggesting that obtaining MI block may be a key mechanism in the salutary effect of EIVOM.

Since VENUS, three recently published prospective randomized controlled trials have uniformly shown a benefit to adjunctive EIVOM combined with RF ablation [12,13,14]. In 2024, Zuo et al. demonstrated in a single-center randomized control trial of 89 patients with persistent AF that adjunctive EIVOM added to an RF ablation strategy consisting of PVI, lateral MI line, roof line, and CTI line substantially increased the likelihood of achieving MI block compared to the same ablation strategy without EIVOM (93.3% vs 64.9%; *p* < 0.05). After one year, 17.8% of patients in the study group had recurrent AF versus 31.8% in the control group. In 2025, Sang et al. reported the results of the Prospective Randomized Comparison Between Upgraded 2C3L Versus PVI Approach for Catheter Ablation of Persistent Atrial Fibrillation (PROMPT-AF) trial [13]. This multicenter randomized control trial compared a strategy of PVI with adjunctive EIVOM and linear ablation of lateral MI, LA roof, and CTI against PVI alone in 495 patients with persistent AF. After 12 months, 70.7% of the intervention group remained free of atrial arrhythmias versus 61.5% of the control group (HR 0.73; *p* = 0.045). Later in 2025, Derval et al. reported results of the Marshall-Plan Ablation Strategy Versus Pulmonary Vein Isolation in Persistent AF trial [14]. This single-center randomized control trial of 120 patients with persistent AF, like PROMPT-AF, compared a strategy of adjunctive EIVOM added to an RF ablation strategy of PVI, lateral mitral line, roof line, and CTI line against PVI alone. At 12 months, 86.4% of patients in the intervention group remained free of AF compared to 66.1% in the control group (*p* = 0.012). Of note, these three trials all used the same lesion set of PVI, roof line, and MI line, variably called the “2 circle 3 line” (2C3L) or “Marshall Plan” lesion set. Taken together with VENUS, a recent meta-analysis of these four trials showed that adjunctive EIVOM increased freedom from atrial arrhythmias (RR 1.21; *p* < 0.01; NNT 10), reduced need for repeat procedures (RR 0.63; *p* < 0.01), and increased likelihood of mitral block (RR 1.30; *p* = 0.03).

These favorable results are not without caveats, however. Notably, while all these trials reached statistical significance, the absolute effect sizes varied considerably, from 9.2% in PROMPT-AF to 20.3% in “Marshall Plan.” While these are important differences, they may not be paradigm-shifting. Additionally, three of the four trials reported failed VOM cannulation in >10% of patients randomized to EIVOM, highlighting the variable patient anatomy and technical challenge inherent in this technique. Moreover, a secondary analysis of VENUS showed that low-volume centers (constituting 10 of the 12 study centers) had markedly less favorable outcomes with adjunctive EIVOM, emphasizing the learning curve associated with this therapy [51]. In addition, secondary analysis of VENUS suggested that favorable outcomes may be mediated by achievement of mitral block, which often requires additional ablation endocardially and epicardially and may not always be achieved. Furthermore, fluoroscopy time was significantly increased and total procedural duration was numerically increased with EIVOM [15]. Finally, while there was no significant difference in major complications, meta-analysis of these trials showed that the overall rate of complications was higher in the EIVOM group, primary driven by pericarditis and pericardial effusion not requiring drainage (RR 2.25; *p* = 0.03). These constraints notwithstanding, the evidentiary support for adjunctive EIVOM in improving AF ablation outcomes appears consistent.

While adjunctive EIVOM has shown favorable outcomes in patients with persistent AF, utilization of this technique is quite low. A recent survey found that only 1% of operators incorporate EIVOM as part of the initial ablation strategy in patients presenting for de novo ablation of persistent AF and only 6.5% of operators employ EIVOM as part of their redo ablation strategy [52]. The reasons for this are likely multifactorial and may include unfamiliarity, as most trainees in electrophysiology are not exposed to chemical ablation, the learning curve, the perceived risk of overall complications, and longer procedural and fluoroscopy times. There also seems to be a concern of widespread myocardial damage attributed to EIVOM. However, an elegant study showed that chemical ablation eliminates voltage over a median of only 3.6% of the LA surface [53].

## 4. Pulsed Field Ablation

### 4.1. Pulsed Field Ablation Mechanism and Comparison to Thermal Ablation of AF

As evidence supporting EIVOM has grown, the advent of pulsed field ablation (PFA) has disrupted the landscape of AF catheter ablation. Rather than using thermal energy, PFA uses ultra-short-duration high-voltage electrical pulses to cause irreversible electroporation of cell membranes, leading to cell death [54]. The waveforms used in energy delivery along with catheter shape and geometry allow for ablation to be performed with myocardial selectivity and minimal thermal energy delivery. While PFA has only been commercially available in Europe since 2021 and in the United States since 2023, there has already been a deluge of data and innovation, with multiple PFA systems already approved for commercial use and more under investigation with numerous catheter designs and features, spanning “single shot” large footprint catheters, focal catheters, catheters with the ability to toggle between PFA and thermal ablation, and more. While comprehensive comparative data encompassing all systems is not yet available in this rapidly changing environment, existing head-to-head studies comparing PFA against thermal ablation have shown non-inferiority of PFA in procedural efficacy, and large real-world registries have demonstrated short procedure times, very low rates of feared major complications such as atrioesophageal fistula, PV stenosis, and phrenic nerve injury, as well as favorable physician learning curves [16,17,18,19,55,56,57,58].

### 4.2. Potential Mechanistic Limitations of Pulsed Field Ablation Compared to EIVOM

Given its acute efficacy, safety and efficiency in PV and posterior LA isolation, electrophysiologists have been quick to leverage PFA in linear ablation at the lateral MI. While dedicated trials directly comparing PFA of the lateral MI and EIVOM are not yet available, existing data suggest that current PFA systems may not replicate the results achieved with EIVOM. First, as mentioned above, a secondary analysis of VENUS demonstrated that favorable outcomes associated with EIVOM were mainly seen in those who achieved durable MI block, indicating that durable MI block may be part of the mechanistic pathway of EIVOM’s salutary effect. While initial studies indicated that acute mitral block was achievable with PFA, later studies have shown that rates of durable MI block are low [59,60,61]. This was perhaps best shown in a recent study by La Fazia et al. In this prospective study of 236 patients undergoing repeat ablation for persistent AF, investigators performed CS isolation, LAA isolation, and MI ablation using an over-the-wire pentaspline PFA system. After a 20 min waiting period, patients received an adenosine challenge to assess for dormant conduction. After a mean of 97 ± 25 days, all patients underwent remapping at the time of a planned staged left atrial appendage occlusion (LAAO) procedure. While acute mitral block was achieved in all patients, after the waiting period and adenosine challenge, 35 patients (14.8%) had recovered MI conduction requiring additional PFA. At the time of the LAAO procedure, remapping revealed durable mitral block in only 13 patients (5.5% of the cohort). In a study of patients with AF undergoing de novo ablation using a focal lattice-tip ablation catheter able to toggle between PFA and RF ablation (RFA), Reddy et al. demonstrated that 100% (78/78) of patients who underwent MI ablation achieved acute mitral block [60]. Importantly, MI block was achieved using a combination of PFA and RFA, and ablation within the CS was also permitted. During protocol-driven remapping performed at a mean of 96 ± 43 days, durable mitral block was seen in 68% of patients. In addition, it should be noted that PFA performed in proximity to coronary arteries has been associated with acute coronary artery vasospasm. Though this may be mitigated with nitroglycerin, the question of possible chronic coronary artery injury remains [62,63]. Based on currently available efficacy and safety data and our experience, it seems likely that dedicated chemical ablation of the VOM with endocardial and epicardial RFA may be required for durable MI block at the present time. Future PFA systems or optimized waveforms may be able to improve lesion durability, and additional studies on the acute and long-term risk of coronary artery injury are needed.

In addition to facilitating MI block, another mechanism by which EIVOM is postulated to improve atrial arrhythmia outcomes is via autonomic denervation. Epicardial atrial ganglionated plexi (GPs), part of the intrinsic cardiac autonomic nervous system, have been implicated in AF initiation and perpetuation in animal and human studies by various mechanisms, including parasympathetically mediated atrial action potential duration shortening as well as sympathetically mediated triggered activity [64,65,66,67]. As mentioned above, the LOM is richly innervated by autonomic nerve fibers and ganglia, which have been shown to contribute to AF initiation and maintenance through atrial ERP shortening and ectopic firing and whose effect can be attenuated by EIVOM. Trials investigating GP ablation as an adjunct to conventional ablation have shown some benefit in meta-analysis, though with notable heterogeneity [68]. GPs are often collaterally ablated during thermal PVI as they are intertwined in the musculature of the PV antra, and this may contribute to some of the beneficial effect of PVI with thermal ablation. In contradistinction, clinical studies have shown that PFA causes substantially less autonomic denervation compared to thermal ablation [69,70]. In an interesting substudy of the ADVENT trial, a prospective randomized trial comparing PFA using a pentaspline PFA catheter against thermal ablation in patients with paroxysmal AF, Gerstenfeld et al. demonstrated that PFA had significantly less effect on autonomic measures such as resting heart rate and heart rate variability change over 1 year compared to thermal ablation [71]. Importantly, this attenuated autonomic denervation did not translate into a difference in arrhythmia outcomes, though this was a one-year outcome study in patients with a paroxysmal phenotype. More recently, Patel et al. presented preliminary data evaluating the efficacy of mitral isthmus ablation with various PFA catheters as judged by elimination of VOM signals recorded from a multipolar catheter placed into the VOM. Linear block was achieved in only a minority of patients with endocardial MI PFA alone. In about 75% of patients, adjunctive alcohol ablation (with additional PFA CS ablation in some) was required to achieve MI block [72].

## 5. Proposed Hybrid Approach

Given the current landscape of available ablation technology and considering the clinical, mechanistic, and safety data above, we have adopted a hybrid strategy of PFA, RFA, and EIVOM in patients with de novo or recurrent persistent AF or perimitral AT. In AF, our strategy involves performing or ensuring PVI followed by posterior wall isolation with PFA. If AF persists, we next consider MI ablation by first mapping for the VOM. If a VOM is present, as it is in about 70–80% of patients, EIVOM is performed [73,74]. EIVOM is followed by linear endocardial RFA at the MI. If conduction across the MI persists, epicardial ablation is performed within the CS with RFA (20 Watts). This approach takes advantage of the efficacy and safety of PFA for PVI and posterior wall isolation and the incremental benefit of EIVOM and RFA in obtaining durable MI block and autonomic modulation, as well as avoiding risk of coronary vasospasm associated with PFA near coronary arteries.

### Technique of Ethanol Ablation of the VOM

**Figure 1:**
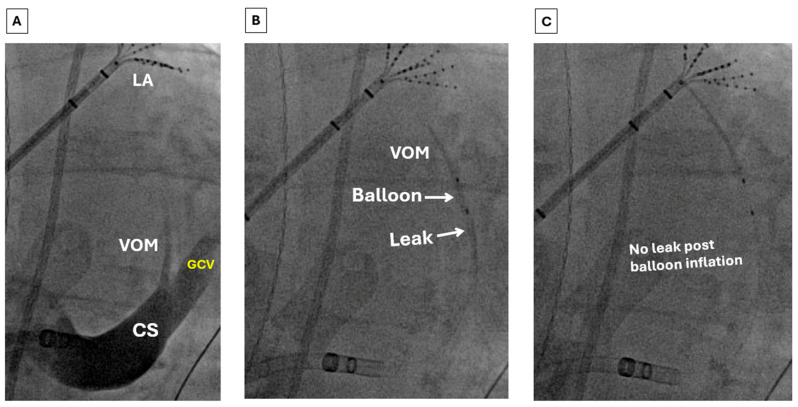
Cannulation of the VOM via an inferior (femoral venous) approach. (**A**) Non-selective venography of the CS via an inferior (femoral venous) approach showing the course of the VOM. (**B**) Initial venography of the VOM through the balloon shows a leak of contrast. (**C**) With inflation of the angioplasty balloon, there is complete occlusion, allowing for the administration of ethanol. Abbreviations: LA = left atrium; VOM = vein of Marshall; CS = coronary sinus; GCV = great cardiac vein.

**Figure 2:**
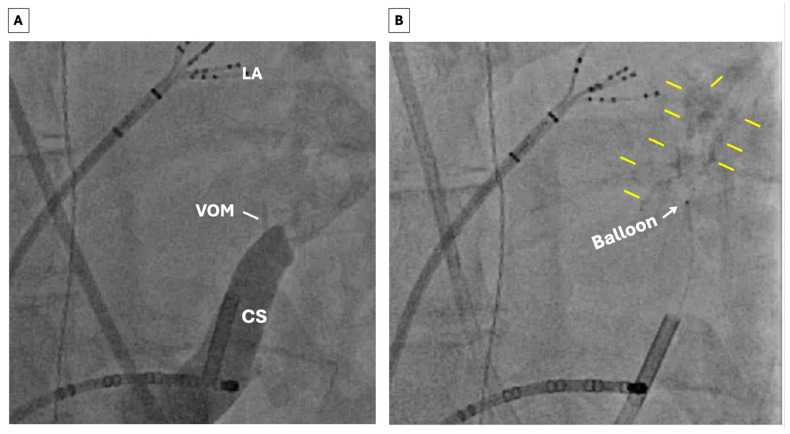
Ethanol infusion into the VOM via an inferior approach. (**A**) Non-selective CS venogram reveals a small VOM. (**B**) Repeat venogram after EIVOM reveals myocardial “blush” outlined by the yellow lines, indicating the extend and locale of the tissue ablated. Abbreviations: LA = left atrium; VOM = vein of Marshall; CS = coronary sinus; EIVOM = ethanol instillation into the vein of Marshall.

**Figure 3:**
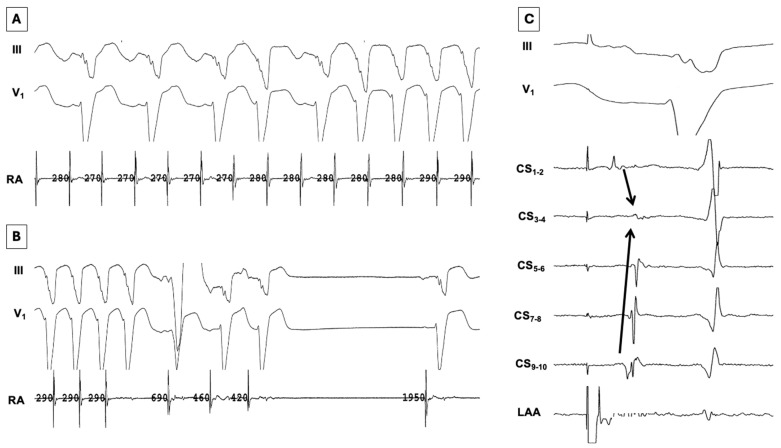
Effect of EIVOM on perimitral flutter. (**A**) After extensive endocardial ablation at the lateral mitral isthmus had failed to terminate perimitral flutter, EIVOM was performed. Progressive slowing of the tachycardia cycle length was observed with eventual 1:1 atrioventricular nodal conduction with a left bundle branch block pattern. (**B**) Further slowing of the tachycardia was observed before termination to sinus rhythm. (**C**) After termination of the tachycardia, pacing from the left arial appendage (LAA) revealed conduction block across the mitral isthmus. Note that there is a collision of wavefronts (arrows) at CS_3-4_, the level of the linear lesion. Abbreviations: RA = right atrium; LAA = left atrial appendage; CS = coronary sinus; EIVOM = ethanol instillation into the vein of Marshall.

Techniques for performing EIVOM have been previously described [75]. The VOM can be accessed either via a superior (internal jugular) approach or an inferior (femoral venous) approach (Figure 1 and Figure 2). For the last several years, we have primarily used the latter approach, reserving the former for patients with variant anatomy. A diagnostic multipolar catheter is first used to cannulate the coronary sinus (CS). From a femoral station, a deflectable sheath is then advanced into the CS over the catheter. The VOM can often be identified with a non-selective venogram of the CS; otherwise, a formal balloon-occlusive venogram can be performed. The VOM is then cannulated with an angioplasty wire, over which a preloaded angioplasty balloon is advanced. The balloon is then inflated to confirm complete occlusion prior to drug delivery (Figure 1, panels B and C). Depending on the clinical scenario, chemical ablation may be performed during sinus rhythm, AF, or perimitral flutter. Epicardial ablation within the CS may be necessary to achieve demonstrable MI block (Figure 3). EIVOM is considered prior to epicardial ablation within the CS as CS ablation has been associated with nonidentification of the VOM [76]. In the case of perimitral reentry persistence despite endocardial MI ablation, EIVOM, and CS ablation, an anterior approach is reasonable, recognizing the risk of slowing conduction into or even electrical isolation of the LAA. Patients in whom the LAA is isolated are considered for LAAO to mitigate risk of thromboembolism. Patients undergoing EIVOM at our center are frequently treated with colchicine to prevent pericardial complications.

## 6. Future Directions

While iterative procedural modifications have been described to improve the success of EIVOM, the development of dedicated tools would be welcome and appear to be under development [77,78,79]. Furthermore, prospective randomized studies are needed that directly assess the incremental benefit of EIVOM in patients undergoing PFA. The Marshall Vein Ethanol Infusion in Addition to Pulsed Electric Field Ablation Versus Pulsed Electric Field Ablation Alone for Paroxysmal Atrial Fibrillation (MARVEL-PAF) trial is a multicenter randomized trial which will evaluate the effective of adjunctive EIVOM in patients with paroxysmal AF and is expected to be completed in 2027 [80]. Additionally, the Posterior Mitral Isthmus Line With Vein of Marshall Ethanolisation Compared With Anterior Mitral Lines in Patients With Persistent Atrial Fibrillation (MIVANT) trial is a randomized control trial of patients with persistent AF which aims to compare PVI-adjunctive lateral MI ablation with EIVOM against a PVI-adjunctive anterior mitral line strategy [75]. Finally, although current waveforms may not reliably create linear lesions across the mitral isthmus, future iterations may be more effective, requiring ongoing re-evaluation of the role of chemical ablation.

## 7. Conclusions

The evidentiary support for adjunctive EIVOM in patients with persistent atrial fibrillation is strong, with several well-designed randomized controlled trials suggesting a consistent moderate effect size in improved outcomes in patients with persistent atrial fibrillation. EIVOM seems to achieve its effect via elimination of rapid electrical activity from the lateral LA, facilitation of durable MI block, and autonomic modulation of atrial tissue. Current-generation PFA systems do not seem to be able to adequately address the arrhythmogenic potential of the LOM, likely owing to its epicardial location, epicardial fat deposition, myocardial selectivity, and risk of coronary artery spasm and injury. In light of this, a hybrid approach incorporating PFA, RFA, and EIVOM may be useful in approaching selected patients with persistent AF. However, as the PFA technology landscape is rapidly changing and considering the technical complexities and added time that EIVOM involves, reassessment of the value of this adjunctive therapy will be necessary in the future.

## Data Availability

No new data were created or analyzed in this study.
